# A new species of *Pristimantis* from southern Ecuador (Anura, Craugastoridae)

**DOI:** 10.3897/zookeys.606.9121

**Published:** 2016-07-21

**Authors:** Paul Székely, Dan Cogălniceanu, Diana Székely, Nadia Páez, Santiago R. Ron

**Affiliations:** 1 Ovidius University Constanţa, Faculty of Natural and Agricultural Sciences, Al. Universităţii, nr. 1, corp B, 900470, Constanţa, Romania; 2 Universidad Técnica Particular de Loja, Departamento de Ciencias Naturales, San Cayetano Alto, calle Marcelino Champagnat s/n, Loja, Ecuador; 3 Universidad Nacional de Loja, CITIAB, Ciudadela Universitaria, La Argelia, EC 110101, Loja, Ecuador; 4 Laboratory of Fish and Amphibian Ethology, Behavioural Biology Unit, University of Liège, 22 Quai van Beneden, 4020, Liège, Belgium; 5 Museo de Zoología, Departamento de Ciencias Biológicas, Pontificia Universidad Católica del Ecuador, Avenida 12 de Octubre 1076 y Roca, Apartado 17–01–2184, Quito, Ecuador

**Keywords:** Anura, Craugastoridae, Pristimantis prometeii sp. n., Reserva Buenaventura

## Abstract

A new species of *Pristimantis* is described from Reserva Buenaventura, southern Ecuador, at elevations between 878 and 1082 m. A molecular phylogeny based on nuclear and mitochondrial genes shows that the new species is closely related to *Pristimantis phoxocephalus*, *Pristimantis riveti*, and *Pristimantis versicolor*. The new species differs from them and other morphologically similar congeners in having a low W-shaped dermal ridge in the scapular region, a large conical tubercle on the upper eyelid and on the heel, a thin mid dorsal fold, and a longitudinal lateral fold starting behind the tympanic fold and extending along the anterior two thirds of the flank. The new species inhabits cloud forests in the Pacific slopes of the Andes.

## Abstract (es)

Describimos una nueva especie de *Pristimantis* de la Reserva Buenaventura, al sur del Ecuador, entre elevaciones de 878 y 1082 m. Una filogenia molecular basada en genes nucleares y mitocondriales revela que la nueva especie está cercanamente relacionada a *Pristimantis phoxocephalus*, *Pristimantis riveti* y *Pristimantis versicolor*. La nueva especie difiere de ellas y otros congéneres morfológicamente similares por presentar un pliegue bajo en forma de “W” en la región escapular, un tubérculo cónico sobre el párpado y en el talón, un delgado pliegue mediodorsal y un pliegue lateral longitudinal que se inicia detrás del pliegue timpánico y se extiende a lo largo de dos tercios del flanco. La nueva especie vive en bosques nublados de las estribaciones pacíficas de los Andes.

## Introduction

The Neotropics have the highest amphibian species diversity in the world, housing almost half the number of known species (Bolaños et al. 2008). This high species diversity is almost entirely endemic, with 96% occurring only in the Neotropics (Bolaños et al. 2008). Our knowledge of Neotropical amphibians is mediocre at best (Duellman 1999), with nearly one-quarter of all known species described over the last decade and 150 to 200 new species described yearly (Catenazzi 2015). A large proportion of South American frogs belong to Terrarana, a clade of direct developing frogs (Hedges et al. 2008; Heinicke et al. 2009) or otherwise known as the superfamily Brachycephaloidea (Padial et al. 2014; Frost 2016). Their eggs are deposited in terrestrial habitats and the embryos develop directly into froglets, bypassing the tadpole stage. Currently this large group that contains more than 1000 species, consists of three families, Brachycephalidae, Craugastoridae and Eleutherodactylidae.

Most craugastorids belong to *Pristimantis* (Jiménez de la Espada 1870), the most speciose genus among terrestrial vertebrates with 494 species (Duellman 1993; Hedges et al. 2008; Frost 2016). Members of this genus, commonly called rain frogs, robber frogs or dirt frogs, are largely restricted to moist, forested habitats in the Andes of Colombia, Ecuador and Peru (Lynch and Duellman 1997; Frost 2016). The taxonomy of these species is challenging because of their high cryptic diversity, intraspecific variation, and the scant morphological characters available to diagnose species (Duellman and Lehr 2009). Despite recent reviews (e.g. Hedges et al. 2008; Padial et al. 2014) the phylogenetic affinities of most species are unknown and numerous new species are discovered and described each year. During the last decade, 125 new species of *Pristimantis* have been described, 30% of which occur in Ecuador (AmphibiaWeb 2016). Just in the past several months, nine new *Pristimantis* species were described from Ecuador (Hutter and Guayasamin 2015; Reyes-Puig et al. 2015; Arteaga et al. 2016; Brito et al. 2016; Navarrete et al. 2016) with probably many more awaiting descriptions. Herein we describe a new species of *Pristimantis* from Reserva Buenaventura, El Oro province, southern Ecuador.

## Materials and methods

### Specimen collection

Field work was carried out between July and September in 2014 and March, April, and July to September in 2015 at several sites in Reserva Buenaventura. The reserve is private and belongs to the Jocotoco Conservation Foundation. The protected area has an altitudinal range between 400 and 1200 m a.s.l. and occurs in a transition zone between Deciduous Costa Forest and Western Montane Forest (*sensu* Ron et al. 2016). We made intensive visual encounter surveys, auditory surveys and leaf litter searches during evenings (18h00–01h00) and also daytime searches in bromeliads. Collected specimens were photographed alive and euthanized using 20% benzocaine, fixed in 10% formalin, and stored in 70% ethanol. Tissue samples that were used for genetic analyses were preserved in 96% ethanol. Examined specimens (listed in the type-series and Appendix 1) are housed in Museo de Zoología de la Pontificia Universidad Católica del Ecuador
(QCAZ).

### Morphology

For the description of qualitative and quantitative morphological characters Duellman and Lehr (2009) was followed. Sex was determined by the presence of vocal slits and/or by gonadal inspection. Color data in life were based on field notes and digital photos. The capitalized colors and their corresponding color codes (in parentheses) used in the color in life descriptions follow Köhler (2012). Measurements were taken with a digital caliper and rounded to the nearest 0.1 mm. All well-preserved specimens were measured for the following morphometric variables: (1) snout-vent length (SVL), distance from tip snout to posterior margin of vent; (2) head width (HW), greatest width of head measured at level of jaw articulation; (3) head length (HL), distance from the tip of snout to posterior angle of jaw articulation; (4) interorbital distance (IOD), distance between the inner margins of the orbits; (5) internarial distance (IND), distance between the inner edges of the narial openings; (6) upper eyelid width (EW), the perpendicular distance to the outer edge of the eyelid; (7) eye diameter (ED), distance between anterior and posterior borders of eye; (8) eye-nostril distance (EN), distance from posterior margin of nostril to anterior margin of eye; (9) tympanum diameter (TD), horizontal distance between peripheral borders of tympanic annulus; (10) femur length (FL), length of femur from vent to knee; (11) tibia length (TL), length of flexed leg from knee to heel; (12) foot length (FoL), distance from proximal margin of inner metatarsal tubercle to tip of Toe IV; (13) hand length (HaL), distance from proximal edge of palmar tubercle to the tip of Finger III.

### DNA extraction amplification and sequencing

DNA was extracted from muscle or liver tissue preserved in 96% ethanol or tissue storage buffer, using standard phenol–chloroform extraction protocols (Sambrook et al. 1989). We used a polymerase chain reaction (PCR) to amplify DNA fragments for the mitochondrial gene 16S rRNA (16S) and the nuclear gene RAG-1, using primers listed in Goebel et al. (1999), Moen and Wiens (2009) and Wiens et al. (2005). PCR amplification was performed under standard protocols and sequenced by the Macrogen Sequencing Team (Macrogen Inc., Seoul, Korea). The newly generated DNA sequences are available on GenBank (Table 1). We also included 12S, 16S and RAG-1 sequences from GenBank. To optimize taxon sampling within *Pristimantis* we carried out a preliminary phylogenetic analysis including all available sequences from GenBank. These analyses showed that the new species was closely related to *Pristimantis phoxocephalus*. Therefore, *Pristimantis phoxocephalus* and closely related species (based on Padial et al. 2014) are included as well as representative species of all major clades within *Pristimantis*. As outgroup we included sequences of *Diasporus*, *Eleutherodactylus*, *Holoaden*, *Hypodactylus*, *Ischnocnema*, *Lynchius*, and *Strabomantis*.

**Table 1.** Voucher and GenBank accession numbers for specimens used in the phylogenetic analysis.

Voucher number	Species	16S	RAG1	12S
MVZ203844	*Diasporus diastema*	EU186682	EU186752	-
USNM314179	*Eleutherodactylus caribe*	EF493385	-	-
USNM327822	*Eleutherodactylus pantoni*	EF493616	-	-
USNM207945	*Holoaden bradei*	EF493366	-	-
MZUSP131872	*Holoaden luederwaldti*	EU186710	-	-
KU178258	*Hypodactylus brunneus*	GQ345248	-	-
ICNMNH23809	*Hypodactylus dolops*	EU368905	-	-
-	*Ischnocnema hoehnei*	EF493359	-	-
USNM318165	*Ischnocnema holti*	EU186722	-	-
KU218210	*Lynchius flavomaculatus*	EU186667	-	-
KU181408	*Lynchius nebulanastes*	EU186704	-	-
KU212327	*Oreobates saxatilis*	EU186708	-	-
USNM286919	*Phrynopus bracki*	EF493709	-	-
KU217786	*Pristimantis acerus*	EF493678	-	EF493678
AJC0573	*Pristimantis achatinus*	JN991420	JQ025168	JN991485
KU217830	*Pristimantis actites*	EF493696	EF493432	EF493696
KU215460	*Pristimantis altamazonicus*	EF493670	EF493441	EF493670
KU177637	*Pristimantis appendiculatus*	EF493524	-	EF493524
KU291638	*Pristimantis bipunctatus*	EF493702	EF493430	EF493702
KU291702	*Pristimantis bromeliaceus*	EF493351	-	EF493351
KU177658	*Pristimantis calcarulatus*	EF493523	-	EF493523
KU217857	*Pristimantis condor*	EF493701	EF493443	EF493701
KU177733	*Pristimantis crucifer*	EU186718	-	EU186736
QCAZ48309	*Pristimantis curtipes*	KX525474	KX525470	-
KU179090	*Pristimantis dissimulatus*	EF493522	-	EF493522
KU217998	*Pristimantis duellmani*	-	EF493438	-
NRPS0055	*Pristimantis erythropleura*	JN991445	JQ025182	JN991509
NRPS0009	*Pristimantis gaigei*	JN991449	JQ025186	JN991513
KU218002	*Pristimantis glandulosus*	EF493676	-	EF493676
KU218015	*Pristimantis inusitatus*	EF493677	-	EF493677
KU218227	*Pristimantis leoni*	EF493684	EF493433	EF493684
MTD45080	Pristimantis cf. mendax	EU186659	-	EU186659
AJC1753	*Pristimantis moro*	JN991453	JQ025192	JN991519
AJC1860	*Pristimantis moro*	JN991454	JQ025191	JN991520
NRPS0048	*Pristimantis nervicus*	JN991456	JQ025194	JN991522
KU177812	*Pristimantis nyctophylax*	EF493526	EF493425	EF493526
KU222023	*Pristimantis ockendeni*	EF493519	EF493434	EF493519
KU218021	*Pristimantis orcesi*	EF493679	-	EF493679
MHNSM9267	*Pristimantis peruvianus*	EF493707	EF493436	EF493707
KU218025	*Pristimantis phoxocephalus*	EF493349	-	EF493349
AJC0594	*Pristimantis pirrensis*	JN991462	JQ025199	JN991528
QCAZ58040	*Pristimantis prometeii*	KX525475	-	-
QCAZ58042	*Pristimantis prometeii*	KX525476	KX525471	-
QCAZ58043	*Pristimantis prometeii*	KX525477	KX525473	-
QCAZ58044	*Pristimantis prometeii*	KX525478	KX525472	-
KU218028	*Pristimantis pycnodermis*	EF493680	-	EF493680
KU218035	*Pristimantis riveti*	EF493348	-	EF493348
KU291635	*Pristimantis sagittulus*	EF493705	EF493439	EF493705
NRPS0085	*Pristimantis savagei*	JN991467	JQ025205	JN991536
KU212220	*Pristimantis schultei*	EF493681	-	EF493681
KU218052	*Pristimantis spinosus*	EF493673	-	EF493673
KU291659	*Pristimantis stictogaster*	EF493704	EF493445	EF493704
KU218147	*Pristimantis subsigillatus*	EF493525	-	EF493525
NRPS0067	Pristimantis aff. taeniatus	JN991429	JQ025171	JN991493
NRPS0001	Pristimantis aff. taeniatus	JN991430	JQ025172	JN991494
USNM336098	*Pristimantis urichi*	EF493699	EF493426	EF493699
KU218096	*Pristimantis versicolor*	EF493389	EF493431	EF493389
KU218116	*Pristimantis walkeri*	EF493518	EF493428	EF493518
ROM43978	*Pristimantis zeuctotylus*	EU186678	-	EU186678
NRPS0060	*Pristimantis zophus*	JN991479	JQ025213	JN991549
CVULA7073	*Strabomantis biporcatus*	GQ345249	-	-
SIUC7062	*Strabomantis bufoniformis*	DQ283165	-	-

The combined DNA matrix had up to 2914 bp. Preliminary sequence alignment was done with MAFFT 7.2 software with the L-INS-i algorithm (Katoh and Standley 2013). The matrix was partitioned to allow independent inferences of models of evolution by gene and by codon position in coding genes. We used software PartitionFinder v. 1.1.1 (Lanfear et al. 2012) to simultaneously estimate both the best-fit model for each partition and the best partition strategy for our data. We defined five a priori partitions (12S, 16S, first, second and third codon position of RAG1). The best partition strategy was selected using the Akaike information criterion (AIC).

### Phylogenetic analysis and genetic distances

Phylogenetic trees were obtained using maximum likelihood searches with software GARLI 2.0 (Zwickl 2006). We made two independent searches with 10 replicates each. The first search started with random trees and the second with stepwise addition trees. We increased the setting “genthreshfortopoterm” until all 10 searches resulted in similar likelihood values, indicating an efficient search. The final setting of “genthreshfortopoterm” was 100,000. Other settings were set to default values. Node support was assessed with 200 pseudoreplicate non-parametric bootstraps (npb; Felsenstein 1985), starting from random trees configured with the same settings of the full search, but with one replicate per run. Uncorrected p-genetic distances were estimated with software Mesquite 2.75 excluding ambiguous sites and gaps (Maddison and Maddison 2011).

## Results

### Phylogeny

**Figure 1.**
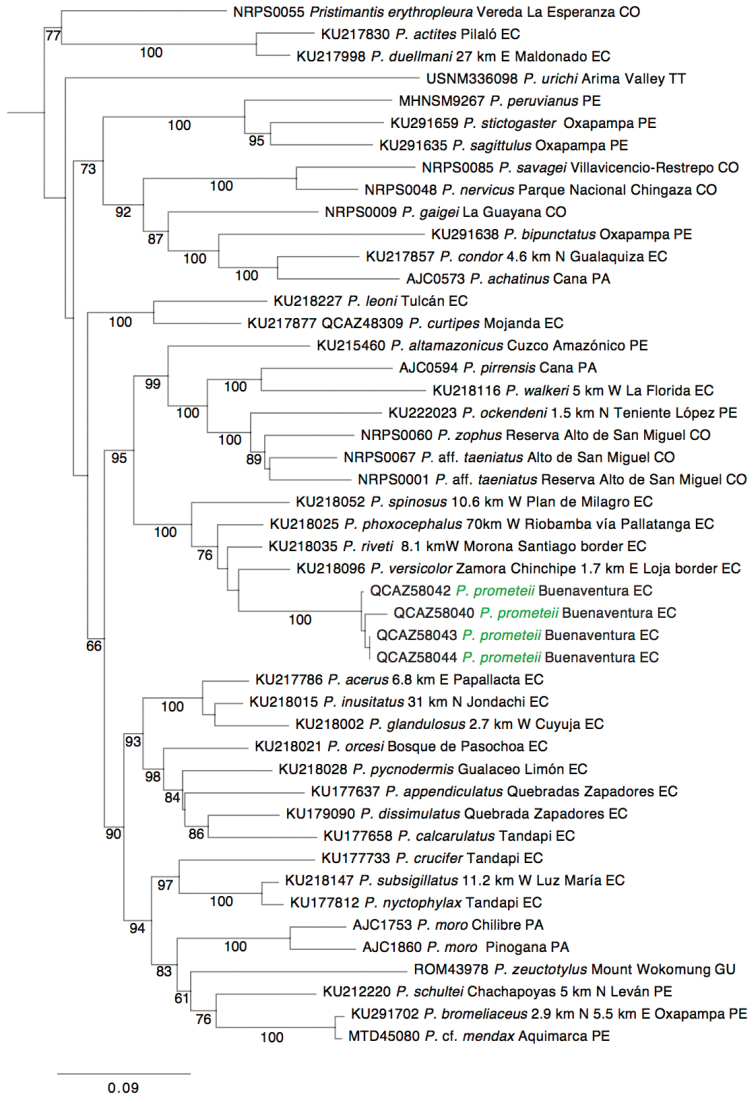
Maximum likelihood phylogram depicting phylogenetic relationships of *Pristimantis prometeii* sp. n. Bootstrap support values are shown under each branch.

The best partitioning scheme consisted of three partitions with their models of evolution in parenthesis: 12S and 16S (GTR + I + G), RAG 1^st^ and 2^nd^ position (HKY + G), and RAG 3^rd^ position (TrNef + G). The phylogeny shows that the new species is most closely related to *Pristimantis versicolor*, *Pristimantis riveti*, *Pristimantis phoxocephalus*, and *Pristimantis spinosus* (Fig. 1). This strongly supported clade is distributed in the Andes of northern Peru and central and southern Ecuador. Uncorrected *p*-genetic distances for the gene 16S between the new species and its closest relative, *Pristimantis versicolor*, range from 0.074 to 0.103. Distances with *Pristimantis phoxocephalus*, *Pristimantis riveti*, and *Pristimantis spinosus* range from 0.075 and 0.130. These large genetic distances and its morphological distinctiveness, clearly demonstrate *Pristimantis prometeii* sp. n. is in fact undescribed. We describe it below.

### Taxonomy

#### Pristimantis prometeii sp. n.

Taxon classificationAnimaliaORDOFAMILIA

http://zoobank.org/EFAA799F-0DE2-4EE2-BDD3-3F22A5B648AB

##### Holotype

**Figure 2.**
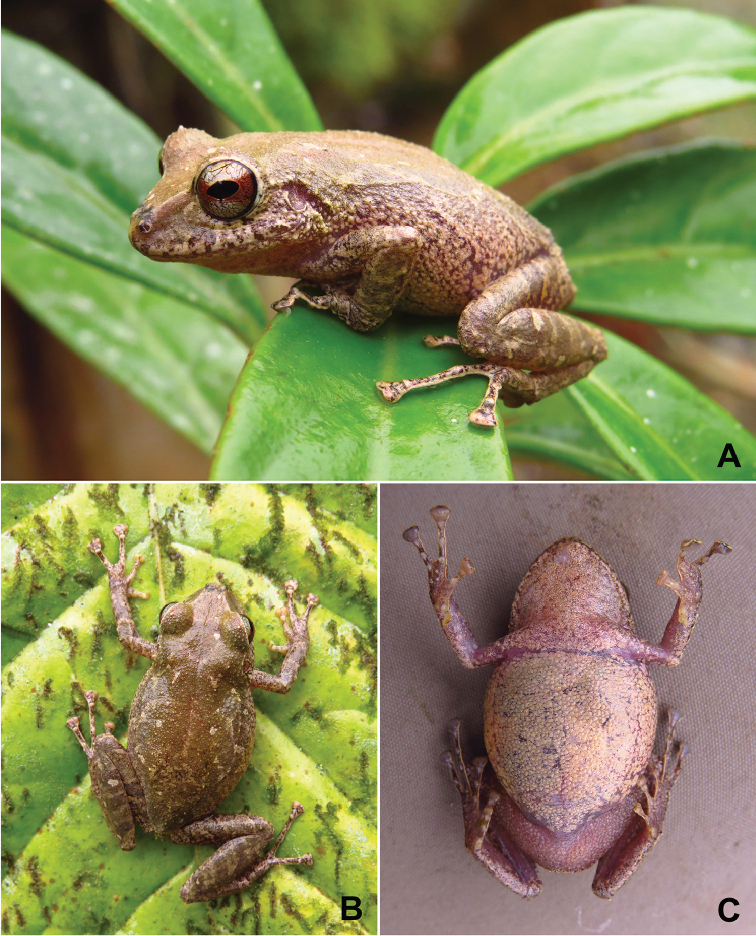
Holotype of *Pristimantis prometeii* sp. n. in life, QCAZ 58044, adult female, SVL 37.6 mm: **A** lateral view **B** dorsal view **C** ventral view.

**Figure 3.**
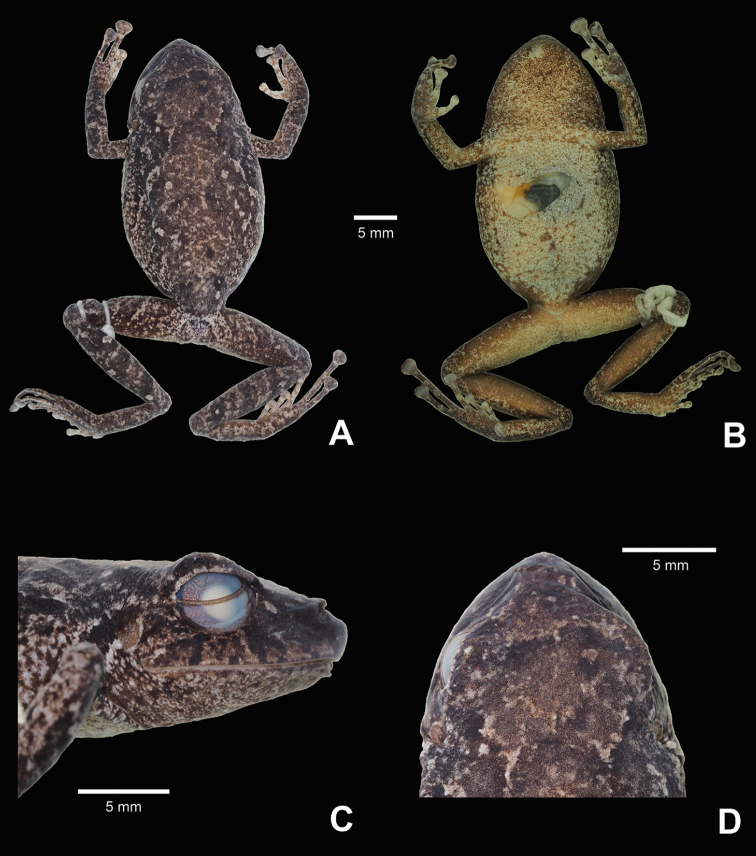
Holotype of *Pristimantis prometeii* sp. n. (QCAZ 58044, adult female) in preservative: **A** dorsal view **B** ventral view **C** head, lateral view **D** head, dorsal view.

**Figure 4.**
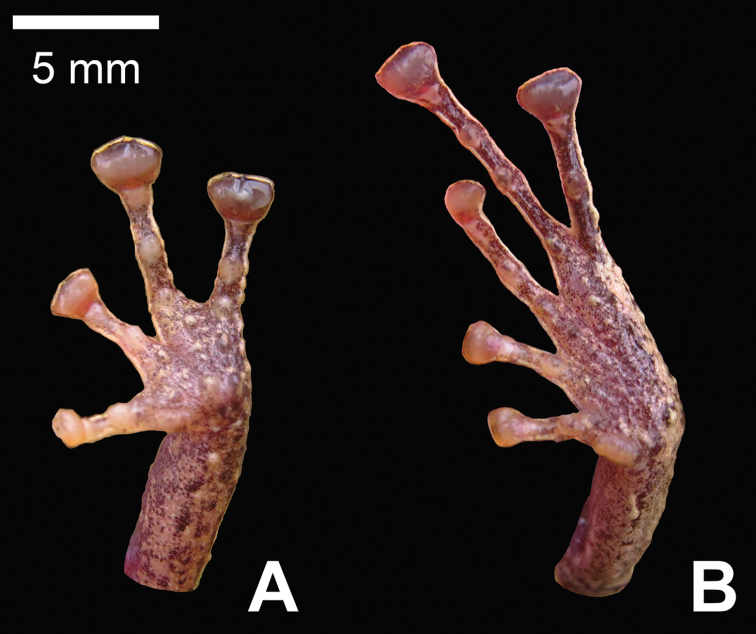
Hand and feet of the holotype of *Pristimantis prometeii* sp. n. in life, QCAZ 58044, adult female: **A** palmar view of hand **B** plantar view of foot.

(Figs 2–4). QCAZ 58044 (field no. SC-PUCE 47291), an adult female from Ecuador, Provincia El Oro, canton Piñas, Reserva Buenaventura, on the reserve’s Sendero del Perico de Orcés (3.6470°S, 79.7565°W; datum WGS84), 878 m above sea level, collected by Dan Cogălniceanu and Paul Székely on 14 September 2014.

##### Paratopotypes.

**Figure 5.**
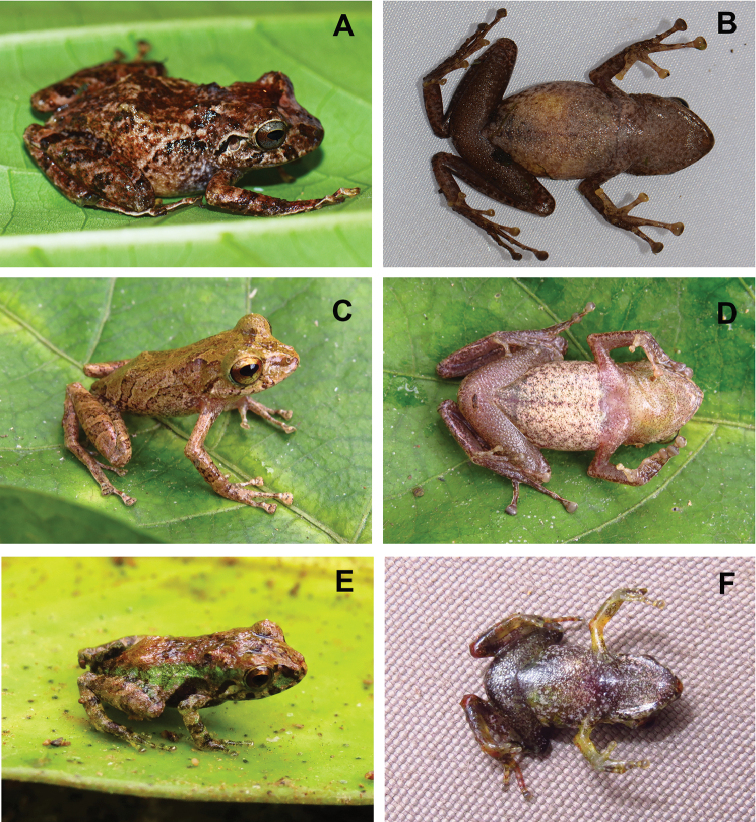
Color variation of *Pristimantis prometeii* sp. n. in life: female paratopotype, QCAZ 62540, SVL 32.6 mm: **A** dorsolateral view **B** ventral view; male paratopotype, QCAZ 58042, SVL 24.9 mm: **C** dorsolateral view **D** ventral view; juvenile, QCAZ 58040, SVL 10.4 mm: **E** dorsolateral view **F** ventral view.

QCAZ 58045 (field no. SC-PUCE 47292), an adult female and QCAZ 58042 (field no. SC-PUCE 47289), an adult male (Fig. 5C, D) collected with the holotype; QCAZ 62540 (field no. SC-PUCE 51624), an adult female (Fig. 5A, B) and QCAZ 62541 (field no. SC-PUCE 51625), an adult male, same data as the holotype but collected by Dan Cogălniceanu on 13 September 2015.

##### Paratypes.

QCAZ 58056 (field no. SC-PUCE 47353), an adult male and QCAZ 58058 (field no. SC-PUCE 47355), an adult female from Ecuador, Provincia El Oro, canton Piñas, Reserva Buenaventura, close to Finca Ramírez (3.6311°S, 79.7618°W), 1082 m above sea level, collected by Dan Cogălniceanu on 7 September 2014; QCAZ 62547 (field no. SC-PUCE 51631), an adult female and QCAZ 62548 (field no. SC-PUCE 51632), an adult male from Ecuador, Provincia El Oro, canton Piñas, Reserva Buenaventura, Quebrada Oscura (3.6652°S, 79.7417°W), 948 m above sea level, collected by Dan Cogălniceanu on 15 September 2015.

##### Additional specimens.

Juveniles, QCAZ 58040 (field no. SC-PUCE 47287) (Fig. 5E, F) and QCAZ 58043 (field no. SC-PUCE 47290) with the same collecting data as the holotype.

##### Diagnosis.

This species is placed in the genus *Pristimantis* based on the general morphological similarity to other members of the genus (e.g. characteristic T-shaped terminal phalanges, toes without membranes, and Toe V longer than Toe III) and based on phylogenetic evidence (Fig. 1). *Pristimantis prometeii* is a medium-sized species distinguished by the following combination of traits: (1) skin on dorsum shagreen with numerous small tubercles; a low W-shaped ridge in the scapular region, usually with four larger warts on it; skin on venter areolate; discoidal fold weak; thoracic fold present; dorsolateral folds absent but with a longitudinal lateral fold from behind the tympanic fold along the 2/3 of the flank length; low mid dorsal fold with rows of small tubercles, especially on the head; (2) tympanic membrane and tympanic annulus prominent, its length about 40% of the length of eye; supratympanic fold obscuring upper and posterodorsal edges of tympanum; (3) snout short, subacuminate in dorsal view, rounded, slightly protruding in profile; canthus rostralis angular; (4) upper eyelid bearing one larger conical tubercle and numerous small tubercles, about 90% IOD in females and 85% IOD in males; cranial crests absent; (5) dentigerous processes of vomers prominent, triangular with 3 to 4 teeth; (6) males with a subgular vocal sac and vocal slits; (7) Finger I shorter than Finger II; discs on fingers broadly expanded, elliptical; (8) fingers bearing broad lateral fringes; (9) ulnar tubercles coalesced into low ulnar fold; (10) heel bearing one larger, conical tubercle and several smaller tubercles; outer edge of tarsus with row of small, conical tubercles; inner edge of tarsus bearing a low fold; (11) inner metatarsal tubercle broadly ovoid, about 5x ovoid outer metatarsal tubercle; supernumerary plantar tubercles present; (12) toes bearing broad lateral fringes; webbing absent; Toe V much longer than Toe III; discs elliptical, about same size as those on fingers; (13) in life, dorsum of various shades of brown, with or without white spots, blotches, or dark brown bars or reticulum; flanks cream, yellow, or green; venter cream with dark flecks and blotches; yellow blotches on the groin, anterior, and posterior surfaces of thighs; iris bronze with fine black reticulations and a median, horizontal read streak; (14) SVL 20.4–24.9 mm in adult males (22.4 ± 1.86 SD, *n* = 4) and 29.9–37.6 mm in adult females (32.7 ± 2.91 SD, *n* = 5).

##### Comparisons with other species.

Comparisons are based on molecular evidence to compare *Pristimantis prometeii* with close relatives and on morphologically similar species present in southern Ecuador and Northern Peru. The phylogenetically closest species are *Pristimantis versicolor*, *Pristimantis phoxocephalus* and *Pristimantis riveti* (Fig. 1). From these three *Pristimantis phoxocephalus* (Lynch 1979) is the most similar. However, it is easily distinguished from *Pristimantis prometeii* by a fleshy vertical keel on the snout. Furthermore, *Pristimantis phoxocephalus* lacks tubercles on the upper eyelid, heel and tarsus, and the low W-shaped dermal ridge in the scapular region. *Pristimantis riveti* (Despax 1911) differs from *Pristimantis prometeii* in having a heel without prominent tubercles (one prominent tubercle in *Pristimantis prometeii*), smaller finger and toe discs, and W-shaped dermal ridge in the scapular region absent. In *Pristimantis versicolor* (Lynch 1979), males lack vocal slits and vocal sacs (both present in *Pristimantis prometeii*), the tarsus lacks distinct tubercles (tubercles present in *Pristimantis prometeii*), lateral fringes are absent in toes (present in *Pristimantis prometeii*), and the dorsum lacks the low W-shaped dermal ridge in the scapular region. Additionally, all these three *Pristimantis* species inhabit upper humid montane forest and subparamo, habitats at higher elevations than those of *Pristimantis prometeii*: 1800-3100 m, in *Pristimantis phoxocephalus* (Lynch and Duellman 1997), 2.620–3.600 m in *Pristimantis riveti* (Coloma et al. 2004) and 2500–3100 m in *Pristimantis versicolor* (Frenkel et al. 2013). The related *Pristimantis spinosus* (Lynch 1979) is also easily distinguished by the presence of cranial crests (absent in *Pristimantis prometeii*), males lacking vocal slits and vocal sacs, and the coloration of groins and concealed surfaces of hind limbs which are black with white spots.

Among the few morphologically similar congeners from southern Ecuador, *Pristimantis sternothylax* (Duellman and Wild 1993) can be distinguished by lacking prominent tubercles on the upper eyelid, having smooth ulnar surfaces, and heel and tarsus lacking tubercles and folds. *Pristimantis buenaventura* (Arteaga et al. 2016) is somewhat similar but it is significantly smaller, and lacks prominent tubercles on the upper eyelid and heel. It also differs by having orange-red spots on the groins. Similar species in northern Peru include *Pristimantis rhodoplichus* (Duellman and Wild 1993), *Pristimantis wiensi* (Duellman and Wild 1993), and *Pristimantis petrobardus* (Duellman 1991). *Pristimantis rhodoplichus* and *Pristimantis petrobardus* differ by lacking prominent tubercles on the upper eyelid. The lack of tympanic membrane readily distinguishes *Pristimantis wiensi* from *Pristimantis prometeii*. Both species also differ in dorsal coloration: green dorsum with scattered bronze and dark blotches in *Pristimantis wiensi* vs. brown dorsum in *Pristimantis prometeii*.

##### Description of the holotype.

Adult female (Fig. 3) with head slightly narrower than body, wider than long, head length 89% of head width, head width 36% of SVL; head length 32% of SVL; snout short (snout to eye distance 14% of SVL), subacuminate in dorsal view, rounded, slightly protruding in profile; canthus rostralis angular; loreal region flat; eye diameter notably greater than eye-nostril distance; nostrils slightly protuberant laterally; lips not flared; cranial crests absent; upper eyelid bearing one larger conical tubercle and numerous small tubercles, width of upper eyelid 94% of IOD; tympanic annulus prominent, round, its upper and posterodorsal part obscured by rounded supratympanic fold; tympanic membrane differentiated, visible; diameter of tympanum 41% of the length of eye; one larger and several low postrictal tubercles situated posteroventrally to tympanic annulus; choanae big, oval, not concealed by palatal shelf of maxillary; vomerine odontophores prominent, triangular, about 3x size of choana, separated medially by distance lower than width of odontophore; each otontophore has 3 to 4 teeth; tongue longer than wide, bilobate, posterior half not adherent to floor of mouth.

Skin on dorsum shagreen with numerous small tubercles; a low W-shaped dermal ridge is present in the scapular region, with 4 larger warts defining its corners (this trait is more visible in life, Fig. 2); thin, low mid dorsal fold starting at tip of snout and ending at cloaca, with rows of small tubercles, especially on the head (trait more visible in life, Fig. 2); dorsolateral folds absent; longitudinal lateral fold from behind the tympanic fold along the 2/3 of the flank length (trait more visible in life, Fig. 2); skin on throat, chest, belly, and ventral surfaces of thighs areolate; discoidal fold weak; thoracic fold present (trait more visible in life, Fig. 2); ornamentation in cloacal region absent.

Ulnar tubercles present, coalescing into low ulnar fold; outer palmar tubercle partially divided distally; thenar tubercle ovoid; subarticular tubercles prominent, round; supernumerary palmar tubercles rounded, smaller than subarticular tubercles; fingers bearing broad lateral fringes; Finger I shorter than Finger II; discs on fingers broadly expanded, elliptical; all fingers bearing pads well defined by circumferential grooves (Fig. 4).

Hind limbs moderately robust; tibia length 46.5% of SVL; foot length 40.7% of SVL; heel bearing one larger, conical tubercle and several smaller tubercles; outer edge of tarsus with row of small, conical tubercles; inner edge of tarsus bearing a low fold; inner metatarsal tubercle broadly ovoid, about 5x ovoid outer metatarsal tubercle; subarticular tubercles prominent, round; plantar supernumerary tubercles rounded, smaller than subarticular tubercles; toes bearing broad lateral fringes; webbing absent; discs on toes elliptical, about same size as those on fingers; toes with ventral pads well defined by circumferential grooves; relative length of toes I <II < III < V < IV; Toe V much longer than Toe III; tip of Toe III not reaching the distal subarticular tubercle on Toe IV; tip of Toe V extending to distal edge of distal subarticular tubercle on Toe IV (Fig. 4).

##### Coloration of holotype.

In life: dorsal background coloration tan (Drab–19), with dirty white spots and blotches of various sizes; flanks cream (Pale Buff–1) with darker reticulum; venter and throat cream (Pale Buff–1) with dark flecks and blotches; dorsal surface of hind limbs with faint darker transverse bars; ventral surfaces of hind limbs salmon (Light Flesh Color–250); groin, anterior and posterior surfaces of thighs with faint yellow (Light Sulphur Yellow–93) blotches; iris bronze with fine black reticulations and a median, horizontal red (Poppy Red–63) streak which is wider at the edges of the eye.

In preservative: dorsal background coloration grayish brown; the white dorsal spots and blotches become more contrasting than in life; venter and throat dirty white with brown flecks and blotches; ventral surfaces of hind limbs brown with white flecks and blotches; the yellow blotches on the groin and anterior and posterior surfaces of thighs visible in life disappear in preservative.

**Measurements of holotype (in mm).** SVL 37.6; head width 13.6; head length 12.1; IOD 3.4; internarial distance 2.9; upper eyelid width 3.2; eye diameter 4.4; eye-nostril distance 3.9; snout to eye distance 5.3; eye to tympanum distance 1.8; tympanum diameter 1.8; femur length 16.8; tibia length 17.5; foot length 15.3; hand length 10.6; Finger I length 5.1. For morphometric variation, see Table 2.

**Table 2.** Measurements (in mm) and morphological proportions (in percentages) of adult males and females of *Pristimantis prometeii* sp. n. (range, average ± SD). Abbreviations for characters are SVL, snout–vent length; HW, head width; HL, head length; IOD, interorbital distance; IND, internarial distance; EW, upper eyelid width; ED, eye diameter; EN, eye-nostril distance; TD, tympanum diameter; FL, femur length; TL, tibia length; FoL, foot length; HaL, hand length.

Character	females (*n* = *5*)	*males* (*n* = *4*)
SVL	29.9–37.6 (32.7 ± 2.91)	20.4–24.9 (22.4 ± 1.86)
HW	10.8–13.6 (12.1 ± 0.99)	7.8–8.5 (8.1 ± 0.33)
HL	9.2–12.1 (10.7 ± 1.07)	5.6–7.8 (6.4 ± 0.99)
IOD	3.1–3.4 (3.2 ± 0.11)	2.2–2.5 (2.3 ± 0.15)
IND	2.2–2.9 (2.5 ± 0.29)	1.3–1.4 (1.4 ± 0.05)
EW	2.8–3.2 (3.0 ± 0.16)	1.7–2.3 (2.0 ± 0.25)
ED	3.9–4.4 (4.1 ± 0.19)	2.5–3.1 (2.8 ± 0.25)
EN	2.9–3.9 (3.5 ± 0.37)	2.4–2.9 (2.6 ± 0.21)
TD	1.4–1.8 (1.6 ± 0.15)	1.0–1.1 (1.1 ± 0.05)
FL	14.7–16.8 (15.7 ± 0.77)	10.3–11.3 (10.8 ± 0.41)
TL	15.3–17.5 (16.7 ± 1.01)	10.8–12.8 (11.9 ± 0.82)
FoL	14.2–15.3 (14.7 ± 0.41)	10.5–11.2 (10.8 ± 0.29)
HaL	8.8–10.6 (9.7 ± 0.66)	5.4–6.9 (6.3 ± 0.65)
HW/SVL	36.1–38.0	34.1–38.2
HL/SVL	30.8–33.7	26.2–31.3
HL/HW	85.2–90.9	71.6–91.8
EN/HL	31.2–35.3	37.2–44.8
ED/HL	36.4–42.4	39.7–48.3
EW/IOD	90.3–94.1	77.3–92.0
EN/ED	74.4–88.6	92.9–96.3
TD/ED	35.9–41.5	35.5–40.7
FL/SVL	44.7–49.2	45.4–50.5
TL/SVL	46.5–53.6	51.4–53.9
FoL/SVL	40.7–47.4	44.9–51.6

**Variation.** Males are smaller than females (Table 2). The dorsal coloration in *Pristimantis prometeii* varies from brown, to green with or without dark brown bars or reticulum. The examined males have dark dorsal bars of various shapes, dark labial bars, dark canthal and supratympanic stripes and on the dorsal surface of hind limbs obvious dark transverse bars (Fig. 5). Males sometimes have a darker reddish-brown (Vinaceous–247) middorsal band, yellow (Sulphur Yellow–80) or greenish (Light Lime Green–113) flanks, and a white or brownish (Tawny–60) interorbital bar. The W-shaped dermal ridge in the scapular region is usually bordered by dark brown or whitish markings, more evident in males than females. The ventral coloration is very similar between females and males, the most important difference being the coloration of the subgular vocal sac in the males, which is yellow (Pale Greenish Yellow–86) with black flecks.

Some females also have the W-shaped scapular dermal ridge dubbed by dark brown coloration and/or labial bars, canthal and supratympanic stripes like the males (Fig. 5). Two juveniles (QCAZ 58040, SVL = 10.4 mm and QCAZ 58043, SVL = 11.5 mm), identified based on the molecular data, have a darker reddish-brown (Kingfisher Rufous–28) middorsal band, green (Apple Green–104) flanks, dark dorsal bars of various shapes, brown (Kingfisher Rufous–28) dorsal surface of the hind limbs with dark transverse bars and present labial bars, canthal and supratympanic stripes (Fig. 5). The low W-shaped dermal ridge in the scapular region, the thin middorsal fold and the incomplete longitudinal lateral fold are also visible. The venter is blackish with white flecks and spots and it is darker than in the adults.

The degree of tuberculation and development of dermal ridges on the dorsum and flanks is usually more evident in males than females. However, the tubercles and dermal folds are difficult to observe in preservative. The low W-shaped dermal ridge in the scapular region, the thin mid dorsal fold, the incomplete longitudinal lateral fold and the thoracic fold are easily observable in life but can be very difficult to notice in the preserved specimens.

##### Etymology.

The specific name is a noun in the genitive case and refers to the Prometeo program of Secretaría de Educación Superior, Ciencia, Tecnología e Innovación, Republic of Ecuador (SENESCYT) through which Dan Cogălniceanu and Paul Székely received funding for their research in southern Ecuador.

##### Distribution and natural history.

**Figure 6.**
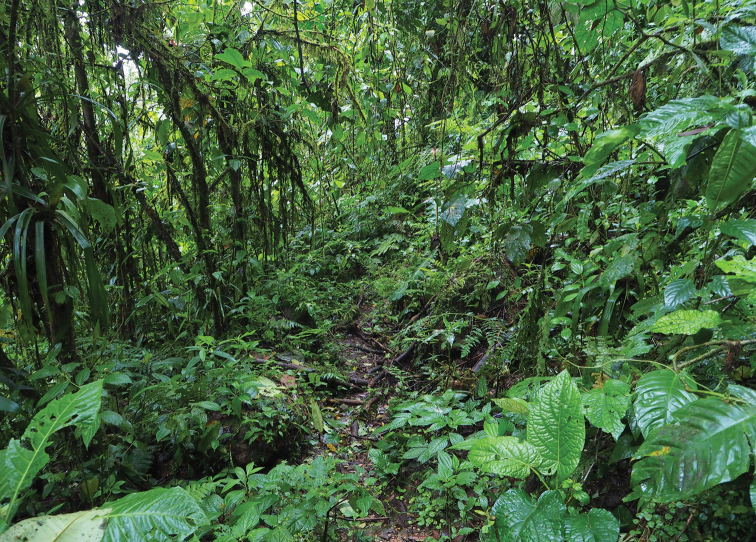
Habitat at the type locality of *Pristimantis prometeii* sp. n. in Reserva Buenaventura.

**Figure 7.**
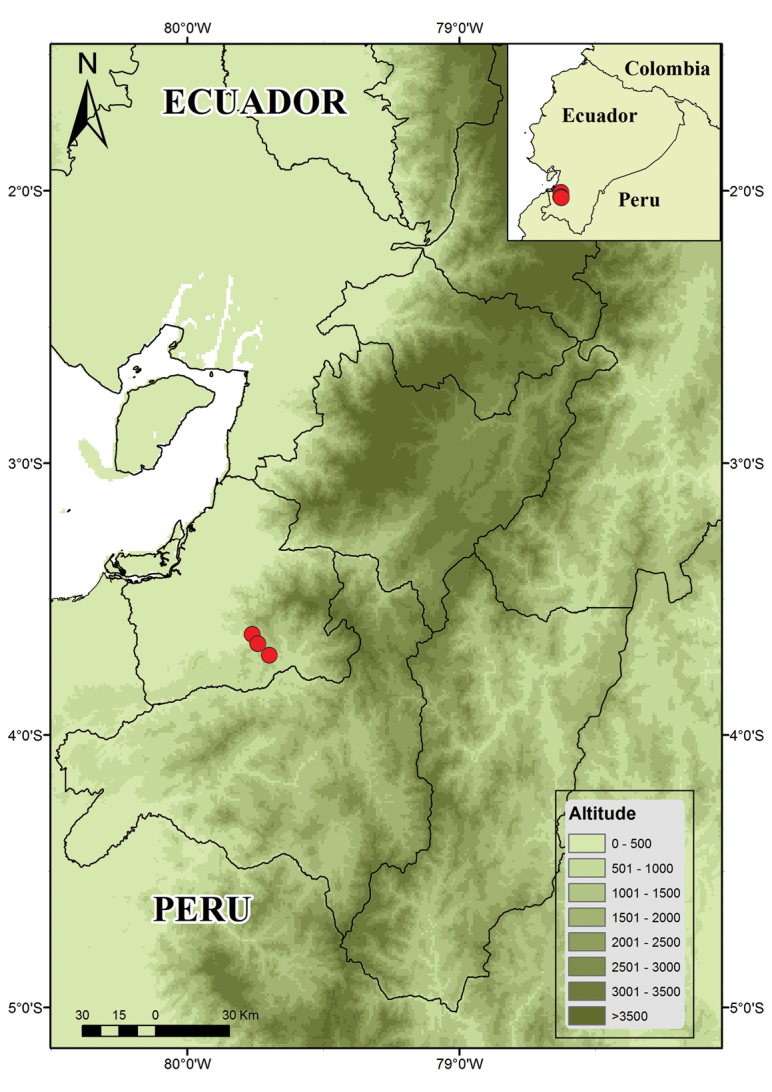
Distribution of *Pristimantis prometeii* sp. n. in Ecuador. Occurrence records are marked with red dots.

*Pristimantis prometeii* is known from three closely located sites at Reserva Buenaventura (Fig. 6), Provincia El Oro, southwestern Ecuador, at elevations between 878 and 1082 m (Fig. 7). Most of the specimens were encountered at night, usually after rains, perching on leaves 10 to 100 cm above the ground. No calling male was found. Two specimens (QCAZ 58056 and QCAZ 58058) were collected during the day in small bromeliads between 2.0 and 2.5 m. All specimens were found in September 2014 and 2015 and additional surveys carried out in 2016 failed to encounter this species. All individuals were found in fairly well-preserved forest areas, near the reserve’s trails or in the vicinity of streams. One of the paratopotypes (QCAZ 58045), an adult female, was missing the right foreleg. Sympatric frog species at the type locality in Reserva Buenaventura include *Pristimantis achatinus* and *Pristimantis subsigillatus* as well as *Epipedobates anthonyi*, *Hyloxalus infraguttatus*, *Espadarana prosoblepon*, *Hypsiboas pellucens* and an undescribed species of *Hyloscirtus*.

##### Conservation status.

*Pristimantis prometeii* sp. n. is only known from three nearby sites in Reserva Buenaventura, Provincia El Oro. Given the scarcity of information on the distribution of the new species, we recommend *Pristimantis prometeii* to be considered as Data Deficient following IUCN’s Red List categories (IUCN 2001).

## Discussion

Our phylogenetic analysis indicates that *Pristimantis prometeii* is most closely related to *Pristimantis versicolor*, *Pristimantis riveti*, *Pristimantis phoxocephalus*, and *Pristimantis spinosus*. The most comprehensive molecular phylogenetic study of terraranas to date also found that these taxa form one clade (Padial et al. 2014). These species were included by Hedges et al. (2008) in the *Pristimantis unistrigatus* group which is the most diverse group of the genus (with almost 200 species), distributed from the lowland Amazon Basin to the high Andes in northeastern South America. This is not a monophyletic group rather it is an assemblage of *Pristimantis* species that do not fit clearly in other groups (Hedges et al. 2008). For this reason, we preferred not to assign *Pristimantis prometeii* to this group until a taxonomic revision will clarify the ambiguous relationships of this large and widely distributed group.

The Reserva Buenaventura was created in 1999 for the protection of two endemic species of birds, and despite its rather small size (about 2400 ha) is an important area for conservation in Southwestern Ecuador. Actually, the reserve is one of the most diverse sites in El Oro province hosting more than 60 species of amphibians and reptiles and 320 bird species (MECN-INB–GADPEO 2015). As for the amphibians, until 2015 there were known 24 species, 22 anurans (from seven families) and 2 caecilians from the reserve (MECN-INB–GADPEO 2015, Yánez-Muñoz et al. 2013, authors’ personal observations). The Craugastoridae family is represented by nine species in the reserve, *Barycholos pulcher* and eight *Pristimanis* species, with several more undescribed ones (authors’ personal observations). The description of this new *Pristimantis* species highlights both the still poor knowledge of amphibians in southern Ecuador and the importance for conservation of even small protected areas, like Reserva Buenaventura, in a constantly degrading environment.

## Supplementary Material

XML Treatment for Pristimantis prometeii

## Appendix I

**Examined specimens**

*Pristimantis cryophilius*

Ecuador–Azuay: Patacocha, vía Gualaceo-Macas (QCAZ 16453); Azuay: Parque Nacional El Cajas, Patul (QCAZ 48677); Morona Santiago: Zuñiag, Galgalan (QCAZ 42513).

*Pristimantis phoxocephalus*

Ecuador–Cotopaxi: Pilaló (QCAZ 556); Alrededores de Pilaló (QCAZ 36846); Pilaló (QCAZ 58465).

*Pristimantis riveti*

Ecuador–Azuay: Parque Nacional El Cajas (QCAZ 7386); El Oro: Chillacocha, 8 km desde Chilla (QCAZ 45157); Chimborazo: vía Pallatanga (QCAZ 20979); Loja: Amaluza–El Salado de Jimbura (QCAZ 30775); Tungurahua: Recinto–Caserío, margen del Parque Nacional LLanganates (QCAZ 46107); Zamora Chinchipe: Reserva Tapichalaca (QCAZ 45677).

*Pristimantis spinosus*

Ecuador–Zamora Chinchipe: Villa Nueva (QCAZ 54002).

*Pristimantis sternothylax*

Ecuador–Loja: 5-10 km de Loja (QCAZ 30600, QCAZ 30602, QCAZ 30604).

*Pristimantis versicolor*

Ecuador–Loja: Reserva Ecológica El Madrigal, Unidad Educativa Amauta (QCAZ 50733); Loja: Shucos (QCAZ 54330); Loja: Vía Yangana-Valladolid (QCAZ 36237).

*Pristimantis walkeri*

Ecuador–Azuay: Recinto La López (QCAZ 53616); Carchi: Cabeceras del Río Baboso al NE de Lita (QCAZ 49514); El Oro: Reserva Ecológica Buenaventura, Río Moromoro (QCAZ 17126); Loja: Cangonamá (QCAZ 49438).
